# Correction: Boorboori, M.R.; Zhang, H.-Y. Arbuscular Mycorrhizal Fungi Are an Influential Factor in Improving the Phytoremediation of Arsenic, Cadmium, Lead, and Chromium. *J. Fungi* 2022, *8,* 176

**DOI:** 10.3390/jof10060376

**Published:** 2024-05-24

**Authors:** Mohammad Reza Boorboori, Hai-Yang Zhang

**Affiliations:** College of Environment and Surveying and Mapping Engineering, Suzhou University, Suzhou 234000, China; m.boorboori@yahoo.com

## Reference Correction

In the original publication [1], Reference Number 52 should be changed to include the following:

52. Nasiri, K.; Babaeinejad, T.; Ghanavati, N.; Mohsenifar, K. Arbuscular mycorrhizal fungi affecting the growth, nutrient uptake, and phytoremediation potential of different plants in cadmium-polluted soil. *Biometals* **2022**, *35*, 1243–1253.

The authors state that the scientific conclusions are unaffected. This correction was approved by the Academic Editor. The original publication has also been updated.
